# Retrieval-extinction of drug memory requires AMPA receptor trafficking

**DOI:** 10.1126/sciadv.add6642

**Published:** 2022-12-23

**Authors:** Xinyou Lv, Junjie Zhang, Ti-Fei Yuan

**Affiliations:** ^1^ Shanghai Key Laboratory of Psychotic Disorders, Brain Health Institute, National Center for Mental Disorders, Shanghai Mental Health Center, Shanghai Jiao Tong University School of Medicine, Shanghai, China.; ^2^ Laboratory Section, Affiliated Tongzhou Hospital of Nantong University, Nantong, China.; ^3^ Co-innovation Center of Neuroregeneration, Nantong University, Nantong, Jiangsu, China.; ^4^ Translational Research Institute of Brain and Brain-Like Intelligence, Shanghai Fourth People’s Hospital Affiliated to Tongji University School of Medicine, Shanghai, China.

## Abstract

Disruption of drug-associated memory reduces relapse. Transient memory retrieval facilitates the upcoming extinction of addiction memory, while the neural basis for this beneficial outcome remains unelucidated. Here, we report that AMPA receptor trafficking acts as the central component for retrieval-extinction–based drug memory intervention. Drug memory retrieval transiently reduces AMPA receptor–mediated synaptic transmission in prefrontal cortical neurons (lasting for 2 to 4 hours) through rapid removal of calcium-permeable AMPA receptors from the synapse, which returned to basal state level after 6 hours. The receptor trafficking is orchestrated by dopamine D1 but not D2 receptor signaling. Blocking AMPA receptor trafficking abolishes retrieval-extinction–mediated addiction memory degradation. These results reveal the molecular mechanism underlying the efficacy of transient memory retrieval on helping to erase addiction memory and support targeting the prefrontal cortex to reduce relapse (e.g., with noninvasive brain stimulation).

## Teaser

Glutamate receptor trafficking mediates drug memory erasure.

## INTRODUCTION

Drug addiction is characterized by its relapsing nature. The persistence of “drug memory” in terms of long-term synaptic plasticity represents a critical cellular mechanism underlying cue-induced drug seeking behavior and the formation of craving. Single drug exposure evokes synaptic transmission changes in midbrain dopamine neurons (*1*), while repeated experiences or self-administration triggers such synaptic modifications in accumbal and cortical regions (*2*, *3*). Eminent efforts have been made to erase the observed synaptic changes using optogenetic or deep brain stimulation–based approaches (*4*–*6*), which accompany the elimination of addiction-related behaviors (e.g., craving and locomotion sensitization) (*7*). However, these manipulations may require invasive procedure to target subcortical structures [e.g., nucleus accumbens (NAc) and paraventricular thalamus], which hinders the therapeutic translation to human subjects.

One effective behavioral therapy for addiction memory erasure is to use the “retrieval-extinction” (R-E) paradigm (*8*). The procedures were first established for fear memory manipulations in both animal and human subjects (*9*, *10*) and then demonstrated to be highly efficient for addiction memory as well (*11*, *12*). Memory retrieval evokes a labile window that allows for reconsolidation disruption, which accompanies series of molecular and cellular events in the memory storage regions (e.g., amygdala for fear memory), including phosphorylation of glutamate receptor subunits and regulation of AMPA receptor (AMPAR)–mediated transmission (*13*). However, the critical brain region and cellular dynamics underlying addiction memory maneuverability following retrieval procedure remain largely unelucidated.

Neuroimaging and neurophysiological evidence emphasizes the importance of the prefrontal cortex (PFC) in cue-evoked neural reactivity and generation of psychological craving (*14*). The extent of cue reactivity in terms of evoked potentials or strength of oscillation [e.g., medial PFC (mPFC) β] could predict the intensity of craving and the risk for relapse (*15*–*17*). In preclinical studies, drug exposure could result in dendritic spine genesis in pyramidal neurons of the prelimbic region in the mPFC (PL-mPFC) (*18*), which parallels the formation of drug-associated memories in vivo (*19*). With considerable evidence that cue or memory retrieval could evoke rapid and notable neural activities in the PFC, the underlying cellular dynamics and its mechanistic link to addiction memory vulnerability are unclear.

Here, we report that R-E of addiction memory requires AMPAR trafficking dynamics in the PFC. The addiction memory formation accompanies the potentiation of AMPAR-mediated synaptic transmission, while memory retrieval procedure triggers the rapid internalization of calcium-permeable AMPARs (CP-AMPARs) within 10 min and lasts for few hours, which is involved in D1 receptor–mediated signaling. Memory reconsolidation accompanies the reinsertion of CP-AMPARs at 6 hours following memory retrieval. Disrupting the receptor dynamics abolished the memory-erasing effects of R-E procedure. These results indicate AMPAR trafficking as critical molecular carriers in addiction memory intervention and support the importance of targeting the PFC to reduce relapse (e.g., with noninvasive brain stimulation).

## METHODS

### Animals

All procedures involving mice were approved by the Institutional Animal Care and Use Committee at Shanghai Mental Health Center, Shanghai Jiao Tong University School of Medicine, and in accordance with the National Institutes of Health guidelines. C57BL/6 mice (8 to 12 weeks old) were housed under a 12-hour light/dark cycle with food and water provided ad libitum.

Brain coordinates of injections were chosen in accordance with the mouse brain atlas: PL-mPFC [anterior-posterior (AP): +1.90 mm; medial-lateral (ML): ±0.40 mm; dorsal-ventral (DV): −2.15 mm]. After surgery, a dummy cannula was inserted, and a cap was screwed on to keep the guide cannula from becoming occluded. Mice were allowed at least 2 weeks to recover before behavioral training commenced.

### Behavioral assay

The behavioral training was performed in a custom-made two-compartment conditioned place preference (CPP) apparatus (42.5 cm by 21.5 cm by 34 cm). Mice were allowed to freely explore both sides of a custom-made CPP training apparatus for 15 min to assess their baseline place preference (pretest). Then, during CPP formation, these mice were injected with saline (intraperitoneally) and confined to their preferred side of the chamber for 45 min and then returned to their home cage. Six hours later, the same mice received an intraperitoneal injection of morphine (15 mg/kg) and were confined to their nonpreferred side of the chamber for 45 min. They were then returned to their home cage. The same training was performed for five consecutive days. Twenty-four hours after the last training session, mice were reexposed to the CPP chamber and allowed to explore both sides of the chamber for 15 min (posttest). CPP scores were calculated by subtracting the time spent in the morphine-paired side of the chamber from the time spent in the other saline-paired side of the chamber. Mice that show an obvious preference (>200 s) during pretest or did not show a strong preference for the morphine-paired chamber (<100 s) during posttest were excluded from further behavioral assays.

The retrieval test was the same as the posttest, except that the duration was 10 min. During extinction or R-E training, saline injections were given in both sides. Extinction training contained 55 min of extinction, while R-E contained 10 min of memory retrieval + 10 min of delay (in the home cage) + 45 min of extinction training for 7 days. The extinction test was the same as the posttest, which was conducted 24 hours after the last extinction training. We injected a low dose of morphine (5 mg/kg, i.p.) to reinstate morphine place preference 24 hours after the extinction test.

### Peptides

The peptide TGL (SSGMPLGATGL) was used to interfere with GluA1 subunit–containing AMPAR trafficking. A similar peptide, AGL (SSGMPLGAAGL), was used as the control for TGL. AGL has a single different amino acid that prevents the peptide from interfering with GluA1 (*3*). The dynamin inhibitory peptide (DIP) (QVPSRPNRAP) was used to block clathrin-mediated endocytosis. A scrambled DIP (QPPASNPRVR) was used as the control of DIP. These peptides were conjugated with a transactivating transcriptional activator (TAT) sequence (GRKKRRQRRRPQ) to facilitate intracellular delivery, resulting in TAT-TGL (GRKKRRQRRRPQSSGMPLGATGL), TAT-AGL (GRKKRRQRRRPQSSGMPLGAAGL), TAT-DIP (GRKKRRQRRRQVPSRPNRAP), and TAT-scramble (GRKKRRQRRRQPPASNPRVR). All peptides were custom-made from GenScript.

### Brain slice electrophysiology

Coronal slices (300 μm thick) containing the mPFC were prepared on a VT1200S vibratome (Leica) in 4°C cutting solution containing 212 mM sucrose, 3 mM KCl, 1.25 mM sodium phosphate buffer, 26 mM NaHCO_3_, 10 mM glucose, and 7 mM MgCl_2_. Slices were incubated in artificial cerebrospinal fluid (ACSF) containing 124 mM NaCl, 2.5 mM KCl, 2 mM MgSO_4_, 2.5 mM CaCl_2_, 1.25 mM sodium phosphate buffer, 22 mM NaHCO_3_, and 10 mM glucose. Slices were placed in the ACSF saturated with 95% O_2_/5% CO_2_ at 34°C for 30 min and then held at room temperature for at least 30 min before experimentation.

During recordings, the slices were placed in a recording chamber constantly perfused with warmed ACSF (28° to 30°C) and gassed continuously with 95% O_2_ and 5% CO_2_. All recordings were made with the γ-aminobutyric acid type A receptor antagonist picrotoxin (100 μM) in the ACSF. Whole-cell recording pipettes (3 to 5 megohms) were filled with a solution containing 120 mM CsMeSO_4_, 20 mM CsCl, 10 mM Hepes, 0.2 mM EGTA, 10 mM sodium phosphocreatine, 5 mM QX314, 4 mM Na_2_ATP, and 0.4 mM Na_2_GTP (pH 7.25 to 7.3; osmolarity, 290 to 299). Data were collected with a MultiClamp 700B amplifier and analyzed by pClamp10 software (Molecular Devices, Sunnyvale, USA). The initial access resistance was <25 megohms and was monitored throughout each experiment. Data were discarded if the access resistance changed by >20% during an experiment. Data were filtered at 2 kHz and digitized at 10 kHz. For the evoked experiments, the stimulating electrode was placed in layer II/III, and layer V pyramidal neurons were recorded. Membrane potential was held at −70 mV to record AMPAR-mediated current and at +40 mV to record *N*-methyl-d-aspartate receptor (NMDAR)–mediated current. Amplitude of NMDAR current was quantified at 50 ms after stimulus, when the contribution of the AMPAR component was minimal.

AMPAR/NMDAR (A/N) ratios were calculated by dividing the amplitude of the AMPAR current (peak current, at −70 mV) by the amplitude of the NMDAR current. To properly assess the rectification of AMPAR excitatory postsynaptic currents (EPSCs), spermine (0.1 mM) was freshly added to the internal solution. The AMPAR current was recorded at three holding potentials (−70, 0, and +40 mV) in the presence of d-aminophosphovalerate (50 μM). Rectification index (RI) of the AMPAR was calculated on the basis of the following equation: RI = [(*I*_0_ − *I*_−70_)/70]/[(*I*_40_ − *I*_0_)/40], in which *I*_−70_, *I*_0_, and *I*_40_ were EPSC amplitudes recorded at −70, 0, and +40 mV, respectively. The paired-pulse ratio (PPR) was calculated by dividing the second evoked EPSC by the first with 20-ms intervals between the two stimulation pulses. AMPAR-mEPSCs were recorded in the presence of tetrodotoxin (1 μM), holding the cells at −70 mV.

For studies involving 1-naphthylacetylsperimine (Naspm), a baseline of 10 min of recording was collected in the presence of ACSF while holding the cell’s voltage at −70 mV. After Naspm (50 μM) had been bath applied to the slice for 30 min to allow sufficient time for blockade of GluA2-lakcing AMPARs, the last 5-min traces were collected to assess the degree of change in AMPAR EPSC peak amplitude.

### Statistics

All results are shown as means ± SEM. All experiments were replicated in 3 to 13 mice. All data collection was randomized. No statistical method was used to predetermine the sample size. All data were analyzed offline, and investigators were blinded to experimental conditions during the analyses.

The Shapiro-Wilk test was used to check the normality of data. Nonparametric Mann-Whitney test or Wilcoxon matched-paired signed-rank test was performed if data were not normally distributed. A paired or unpaired two-tailed Student’s *t* test was conducted for the statistical comparisons of data between two groups. One- or two-way analysis of variance (ANOVA) was conducted for the statistical evaluation of data among more than two groups. Geisser-Greenhouse correction was applied to the data to ensure equal variability of difference in ANOVA, which was followed by Bonferroni post hoc test for multiple comparisons between groups. Statistical significance was set at *P* < 0.05 for all experiments. Statistical analyses were performed in GraphPad Prism (v.8).

## RESULTS

### R-E procedure prevents reinstatement and modifies PL synaptic strength

**Fig. 1.**
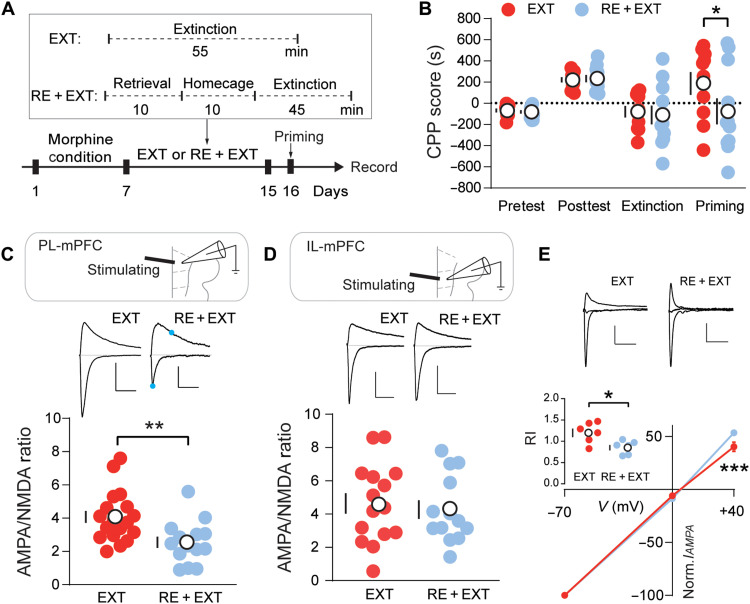
R-E procedure prevents reinstatement and modified PL synaptic strength. (**A**) Experimental timeline of behavior and recording. (**B**) Effect of the experimental manipulations on the CPP score shows that R-E procedure prevented drug priming–induced reinstatement of morphine CPP (EXT/RE + EXT, *n* = 10/11). Two-way ANOVA [*F*(1, 76) = 2.153, *P* < 0.05] followed by post hoc Sidak’s test. *P < 0.05. (**C**) Schematic of recording (top), example traces (middle), and quantification (bottom) of evoked EPSCs at −70 and +40 mV shows that R-E procedure significantly reduced AMPA/NMDA ratio in the PL-mPFC (EXT/RE + EXT, *n* = 18/14). Unpaired *t* test. **P < 0.01. For comparison, EPSC amplitudes are normalized to peaks at +40 mV. Solid dots indicate the current amplitude used for calculating the A/N ratio. Scale bars, 100 pA and 50 ms. (**D**) Schematic of recording (top), example traces (middle), and quantification (bottom) of evoked EPSCs at −70 and +40 mV shows that R-E procedure did not affect AMPA/NMDA ratio in the IL-mPFC (EXT/RE + EXT, *n* = 15/13). Unpaired *t* test. *P* > 0.05. Scale bars, 100 pA and 50 ms. (**E**) Example traces (top), current-voltage (*I*/*V*) curve (bottom), and quantification (inset) of evoked AMPAR EPSCs at −70, 0, and +40 mV show that rectification was abolished in the RE + EXT group (EXT/RE + EXT, *n* = 6/6). Two-way ANOVA [*F*(1, 30) = 3.697, *P* < 0.001] followed by post hoc Sidak’s test. ***P < 0.001. Unpaired *t* test. *P < 0.05. Scale bars, 50 pA and 50 ms. EXT, extinction; RE + EXT, retrieval + extinction. Means ± SEM.

We first established the mouse model of addiction memory reinstatement using morphine place preference protocol (Fig. 1A). When compared to extinction alone, R-E procedure efficiently prevented priming-induced reinstatement (Fig. 1B). To investigate the potential cellular mechanisms differentiating extinction alone and R-E procedure, we performed ex vivo brain slice recording on different regions [prelimbic (PL) cortex and infralimbic (IL) cortex] of the PFC of mice from both groups. There was a significant reduction in A/N ratio in PL-mPFC layer V pyramidal neurons in the R-E group when compared to the E group but not for IL-mPFC (Fig. 1, C and D). The reduced AMPAR transmission was also reflected by the reduction of amplitude but not frequency of mEPSC in the R-E group of PL-mPFC region neurons (fig. S1, A to C). To further dissect the subunit composition changes, we measured the current-voltage relationships of evoked EPSCs. RI analysis suggested that the AMPAR EPSCs in PL-mPFC neurons from the R-E group contained reduced CP-AMPAR components (Fig. 1E), in line with decreased sensitivity of AMPAR EPSCs to bath application of Naspm, a selective antagonist of CP-AMPAR (fig. S1D). These results indicated that the R-E procedure efficiently erased addiction memory, accompanying selective synaptic strength modification in the PL region.

### Memory retrieval but not extinction procedure mobilizes AMPARs and evokes synapse remodeling

**Fig. 2.**
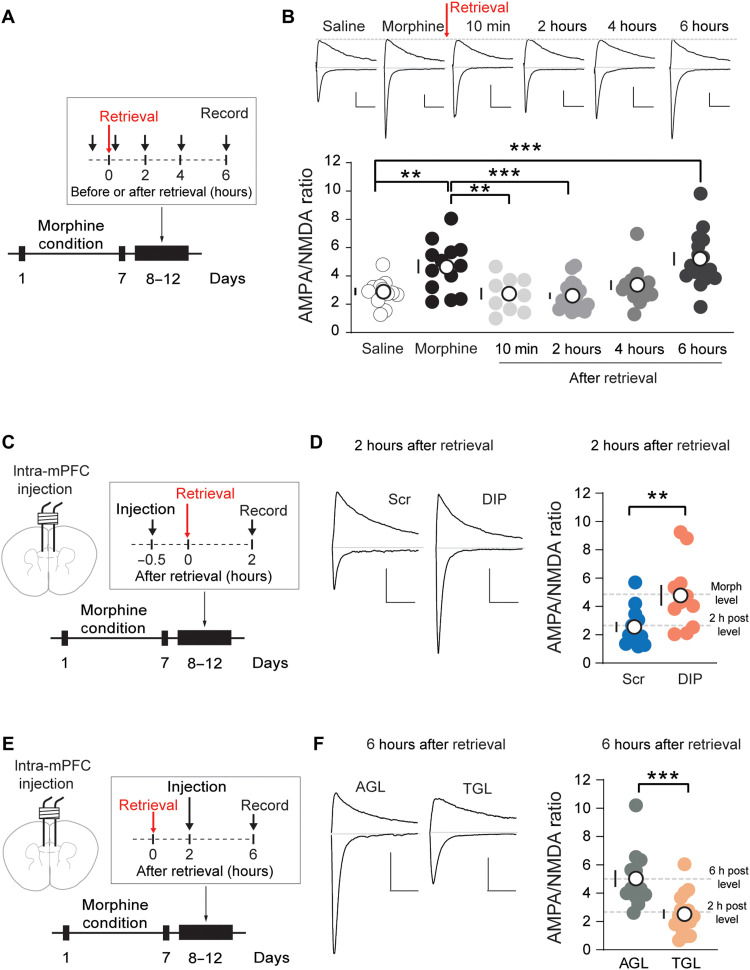
Memory retrieval mobilizes CP-AMPARs and mediates synapse remodeling. (**A**) Experimental timeline. (**B**) Example traces (top) and quantification (bottom) of evoked EPSCs at −70- and +40-mV recording from the saline; morphine (without retrieval); and 10-min, 2-hour, 4-hour, and 6-hour postretrieval groups show an increase in AMPA/NMDA ratio after morphine condition and a quick decrease and recovery ~6 hours after retrieval (saline/morphine/10 min/2 hours/4 hours/6 hours, *n* = 12/13/9/19/14/16). One-way ANOVA [*F*(5, 77) = 1.875, saline versus morphine, *P* < 0.01; saline versus 6 hours, *P* < 0.001] followed by Dunnett’s test. One-way ANOVA [*F*(5, 77) = 1.875, morphine versus saline, *P* < 0.01; morphine versus 10 min, *P* < 0.001; morphine versus 2 hours, *P* < 0.001] followed by Dunnett’s test. **P < 0.01 and ***P < 0.001. Scale bars, 50 pA and 50 ms. (**C**) Experimental timeline of endocytosis prevention manipulation. (**D**) Example traces (left) and quantification (right) of evoked EPSCs at −70- and +40-mV recording 2 hours after retrieval show that DIP suppressed retrieval-induced synapse remodeling (scramble/DIP, *n* = 13/11). Unpaired *t* test. **P < 0.01. Scale bars, 100 pA and 50 ms. Morph level, morphine level [data from (B)]; 2 h post level, 2 hours after retrieval [data from (B)]; Scr, scramble peptide. (**E**) Experimental timeline of reinsertion prevention manipulation. (**F**) Example traces (left) and quantification (right) of evoked EPSCs at −70- and +40-mV recording 6 hours after retrieval show that intra-mPFC TGL peptide injection 2 hours after retrieval suppressed the recovery of synapse (AGL/TGL, *n* = 12/17). Unpaired *t* test. ***P < 0.001. Scale bars, 100 pA and 50 ms. 2 h post level, 2 hours after retrieval [data from (B)]; 6 h post level, 6 hours after retrieval [data from (B)]. Means ± SEM.

We therefore focused on the PL region and examined the potential effects of memory retrieval procedure on AMPAR dynamics. We performed memory retrieval procedure and recorded the AMPAR-mediated synaptic transmission in the PL region (Fig. 2A). We found that repeated morphine exposure induced potentiation of AMPAR transmission, reflected by A/N ratio increase when compared to control animals (Fig. 2B), with unaffected PPR as the presynaptic release parameter between the two groups (fig. S2, A and B). Ten minutes following memory retrieval, there was a rapid and significant decrease in A/N ratio, which remained low at 2 and 4 hours but returned to high level at 6 hours following memory retrieval (Fig. 2B). Such retrieval-induced suppression of AMPAR transmission accompanied the altered RI (figs. S2, D to F, and S3, A to C) and Naspm sensitivity of AMPAR EPSCs (figs. S2, G to I, and S3D), indicating the dynamics of CP-AMPARs in retrieval-induced synaptic remodeling. In addition, the observed retrieval-induced synaptic remodeling also occurs in female mice (fig. S4).

To detect whether extinction alone (without retrieval) induces a similar pattern of AMPAR trafficking, we recorded the AMPAR-mediated synaptic transmission 2 hours after a single extinction session. The results showed that extinction alone did not alter the synaptic strength, indicating that retrieval procedure is required for the initiation of AMPAR trafficking (fig. S3, E and F).

We further investigated the molecular substrate underlying CP-AMPAR trafficking. We locally infused guanosine triphosphatase DIP that blocks clathrin-mediated endocytosis (*20*) or scramble peptide (50 μM, per side) into the PL-mPFC 30 min before retrieval and recorded synaptic strength 2 hours after retrieval (Fig. 2C). DIP treatment prevented retrieval-induced synaptic weakening and the removal of CP-AMPARs from synapse (Fig. 2D). On the other hand, infusion of the small peptide TGL that binds to the C terminus of GluA1 (*3*) into the PL-mPFC 2 hours after retrieval could prevent the reinsertion of CP-AMPARs at PL synapses during reconsolidation (Fig. 2, E and F).

### AMPAR trafficking is required for R-E–mediated memory erasure

**Fig. 3.**
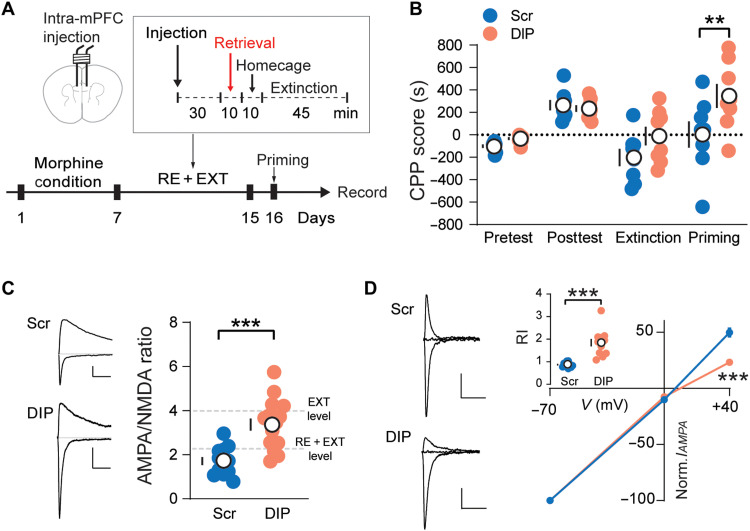
Blocking retrieval-induced AMPAR trafficking abolished R-E–mediated drug memory degradation and synaptic strength modification in PL-mPFC. (**A**) Experimental timeline of behavior and recording. (**B**) Effect of the experimental manipulations on the CPP score shows that the intervention efficacy of R-E procedure on reinstatement was abolished in the DIP group (Scr/DIP, *n* = 8/8). Two-way ANOVA [*F*(1, 56) = 9.409, *P* < 0.01], followed by post hoc Sidak’s test. **P < 0.01. (**C**) Example traces (left) and quantification (right) of evoked EPSCs at −70 and +40 mV show that the intervention efficacy of R-E procedure on synapse was abolished in the DIP group (Scr/DIP, *n* = 12/16). Unpaired *t* test. ***P < 0.001. Scale bars, 50 pA and 50 ms. (**D**) Example traces (left), *I*/*V* curve (right), and quantification (inset) of evoked AMPAR EPSCs at −70, 0, and +40 mV show that the DIP blockade abolished rectification in the PL-mPFC (Scr/DIP, *n* = 10/12). Two-way ANOVA [*F*(1, 60) = 25.54, *P* < 0.001] followed by post hoc Sidak’s test. ***P < 0.001. Unpaired *t* test. ***P < 0.001. Scale bars, 50 pA and 50 ms. Means ± SEM.

To further examine the behavioral relevance of retrieval-induced AMPAR trafficking, we infused DIP or scramble peptide in the PL-mPFC 30 min before retrieval in the R-E procedure daily for 7 days (Fig. 3A). Preventing AMPAR endocytosis by DIP peptide infusion did not directly affect CPP expression (fig. S5, A and B), while combining DIP and retrieval efficiently abolished the R-E–mediated memory erasure in the reinstatement test (Fig. 3B), when compared to the scramble peptide group, accompanied with the preservation of the synaptic potentiation status (higher A/N ratio and CP-AMPAR component; Fig. 3, C and D, and fig. S6). These results suggested that AMPAR trafficking was required for R-E procedure–mediated modification of PL synaptic strength and memory erasure.

We then blocked the reinsertion of CP-AMPARs and assessed the potential effects on addiction memory. Mice that went through morphine condition and extinction training were used in this experiment. Following the retrieval procedure as described before, TGL peptide was infused into the PL-mPFC 2 hours after retrieval to suppress synapse recovery (fig. S7A). We found that the synaptic strength was maintained at a low level in the TGL group, while the AGL group returned to strong level at 6 hours after retrieval (fig. S7B). At the behavioral level, when a low-dose morphine was injected to induce priming (fig. S7C), we found that TGL-treated mice exhibited significantly decreased reinstatement, when compared to AGL-treated mice (fig. S7D). These data indicated that preventing the recovery of remodeling synapse compromises the reconsolidation of addiction memory.

### D1 receptor signaling regulates retrieval-induced synapse remodeling

**Fig. 4.**
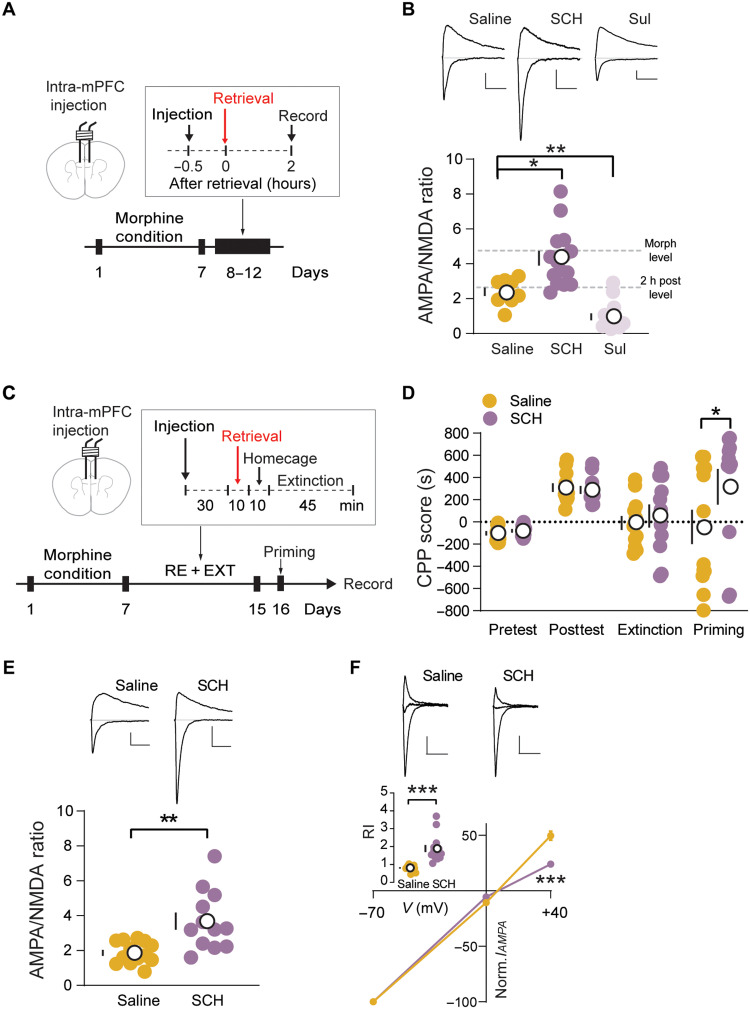
Memory retrieval recruits dopamine D1 receptor signaling to mediate synapse remodeling. (**A**) Experimental timeline of intra-mPFC injection and recording. (**B**) Example traces (top) and quantification (bottom) of evoked EPSCs at −70- and +40-mV recording 2 hours after retrieval show that the dopamine D1 receptor antagonist SCH23390, but not D2-like receptor antagonist sulpiride, suppressed retrieval-induced synapse remodeling (saline/SCH/Sul, *n* = 9/15/15). One-way ANOVA [*F*(2, 36) = 30.99, saline versus SCH, *P* < 0.001; saline versus Sul, *P* < 0.05] followed by Dunnett’s test. *P < 0.05 and **P < 0.01. Scale bars, 50 pA and 50 ms. Morphine level (data from Fig. 2B); 2 h post level, 2 hours after retrieval (data from Fig. 2B). SCH, SCH23390; Sul, sulpiride. (**C**) Experimental timeline of behavior and recording. (**D**) Effect of the experimental manipulations on the CPP score shows that the intervention efficacy of R-E procedure on reinstatement was abolished in the SCH23390 group (saline/SCH, *n* = 12/11). Nonparametric Mann-Whitney *U* test, *P < 0.05. (**E**) Example traces (top) and quantification (bottom) of evoked EPSCs at −70 and +40 mV show that the intervention efficacy of R-E procedure on synapse was abolished in SCH23390 (saline/SCH, *n* = 13/12). Unpaired *t* test. **P < 0.01. Scale bars, 50 pA and 50 ms. (**F**) Example traces (top), *I*/*V* curve (bottom), and quantification (inset) of evoked AMPAR EPSCs at −70, 0, and +40 mV show rectification in the SCH23390 group (saline/SCH, *n* = 13/12). Two-way ANOVA [*F*(1, 81) = 13.86, *P* < 0.001] followed by post hoc Sidak’s test. ***P < 0.001. Unpaired *t* test. ***P < 0.001. Scale bars, 50 pA and 50 ms. Means ± SEM.

Drug-associated cue retrieval induces dopamine release in the PFC (*21*). Therefore, we further investigated the potential involvement of dopaminergic signaling in the regulation of AMPAR trafficking. We infused either the D1 receptor antagonist SCH23390 (4 μg per side) or D2 receptor antagonist sulpiride (4 μg per side) or saline into the PL-mPFC 30 min before retrieval and recorded synaptic strength 2 hours after retrieval (Fig. 4A). The results showed that blocking D1 receptor signaling abolished the synaptic weakening, while blocking D2 receptor facilitated the process (Fig. 4B). We then performed daily SCH infusion into the PL-mPFC in the R-E procedure daily for 7 days (Fig. 4C). We found that SCH treatment did not directly affect CPP expression (fig. S8), while combining SCH treatment and memory retrieval efficiently eliminated the R-E–induced memory erasure (Fig. 4D). This is accompanied with the preservation of CP-AMPAR components in the SCH-treated group (Fig. 4, E and F, and fig. S9). These results emphasized the critical role of dopamine signaling in R-E–mediated synaptic remodeling and memory erasure.

## DISCUSSION

The present study reports that retrieval of drug memory induces rapid and transient depression of synaptic strength in the PL-mPFC, which is mediated through the endocytosis of CP-AMPARs from the membrane. The process is initiated with dopamine release in the PFC region and mediated by D1 receptor signaling. Preventing AMPAR endocytosis abolished the memory erasure effects of R-E procedure. These findings support the importance of AMPAR trafficking as critical targets for addiction memory intervention and highlight the therapeutic value in stimulating the PL cortex [human brain region analog as dorsolateral PFC (DLPFC)] (e.g., with noninvasive brain stimulation) to reduce relapse.

R-E procedure has been proven to be effective for relapse prevention on a variety of preclinical animal models and human patients (e.g., heroin and nicotine) (*12*, *22*). Apart from extinction training, direct protein synthesis inhibition or β-adrenergic receptor inhibition could also induce weakening of drug memory (*11*, *23*). The present results pointed out that AMPAR trafficking acts as the critical cellular component following drug memory retrieval (*3*), and it will be interesting to dissect the potential effects of protein synthesis inhibition or β-adrenergic receptor signaling on AMPAR dynamics in the future.

Notably, here, we found that dopaminergic signaling orchestrates AMPAR trafficking in the PL cortex. D1 receptor signaling is involved in bidirectional modulation of AMPAR trafficking in the mPFC during both long-term potentiation and long-term depression (*24*). Direct activation of a D1 receptor–cyclic adenosine monophosphate–dependent protein kinase pathway by G_s_ protein seems to play an important role in the process, gated by the precise release of dopamine in the PFC (*25*). One potential source of dopamine release is the ventral tegmental area (VTA)-mPFC projection, and the circuitry mechanism associating memory retrieval and VTA activation yet requires future investigation. Nevertheless, the current findings support the use of dopaminergic manipulation (e.g., D1 receptor agonism) in combination with psychological therapies (e.g., memory retrieval) for addiction memory intervention.

Our findings indicate the PL cortex as one critical neural substrate underlying addiction memory retrieval and reconsolidation. Previous studies reported that the PL-mPFC neurons are activated in response to drug-associated cues (*26*, *27*) and exhibited spine increases following addiction memory formation (*18*). In addition, PL-NAc circuit is strengthened following cocaine self-administration, while optogenetic long-term depression of PL-NAc synapses prevented incubation of craving (*28*). On the other hand, the IL-mPFC was demonstrated to be involved in drug seeking (*29*, *30*) and inhibiting conditioned responses after extinction (*28*, *31*). In the present study, we did not conduct further investigation of IL because the synaptic strength between the E group and the R-E group showed no difference. Future studies are required to systemically examine the potential involvement of PL and IL during memory retrieval and reconsolidation process (e.g., with calcium imaging on live animals).

In conclusion, our results indicate AMPAR trafficking in the PL cortex as the critical cellular mechanism underlying retrieval and extinction of addiction memory. These findings support combining memory retrieval and brain stimulation treatment in addiction medicine.

## Notes

View/request a protocol for this paper from *Bio-protocol*.
