# Supplementary material for: Retrieval-extinction of drug memory requires AMPA receptor trafficking
Source: Sci Adv. 2022 Dec 23;8(51):eadd6642. doi: 10.1126/sciadv.add6642 (PMC9788760; doi:10.1126/sciadv.add6642)
Supplement: Supplementary file 1 — Figs. S1 to S9 [file sciadv.add6642_sm.pdf]

Science Advances
AAAS

Supplementary Materials for

Retrieval-extinction of drug memory requires AMPA receptor trafficking

Xinyou Lv *et al.*

Corresponding author: Ti-Fei Yuan, ytf0707@126.com

Sci. Adv. **8**, eadd6642 (2022)
DOI: 10.1126/sciadv.add6642

This PDF file includes:

Figs. S1 to S9

Supplementary data

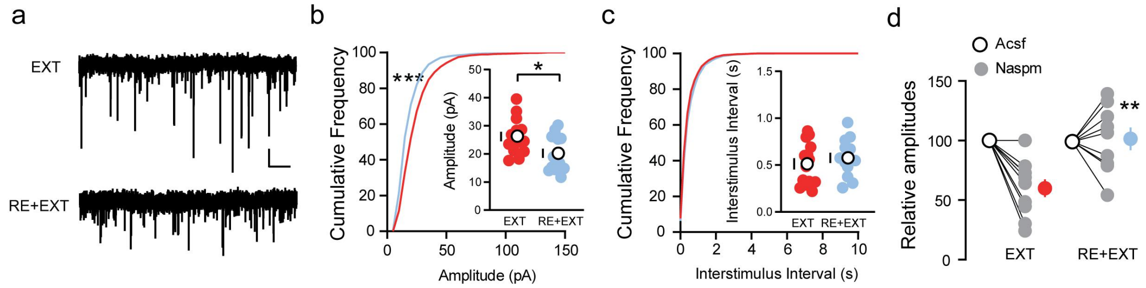

Figure S1. Retrieval-extinction procedure reduced postsynaptic AMPARs in PL-mPFC. a-c, Example traces (a) and mean and cumulative frequency (b,c) of mEPSCs show retrieval-extinction procedure reduced amplitude but leave frequency unchanged (EXT/RE+EXT, n=13/13). The mean amplitude, unpaired t test. *$p < 0.05$; the mean interstimulus interval of mEPSCs, unpaired t test. $p > 0.05$; cumulative frequency of amplitude, Kolmogorov-Smirnov test, ***$p < 0.001$; cumulative frequency of interstimulus interval, Kolmogorov-Smirnov test, $p > 0.05$. Scale bar: 10 pA, 1 s. d, Quantification of EPSCs before (black outlined) and during (gray filled) perfusion of Naspm (50 μM) show a significantly decreased presence of synaptic CP-AMPAR in RE+EXT group (EXT/RE+EXT, n=10/9). Unpaired t test. **$p < 0.01$. EXT, extinction; RE+EXT, retrieval+extinction. Mean ± s.e.m.

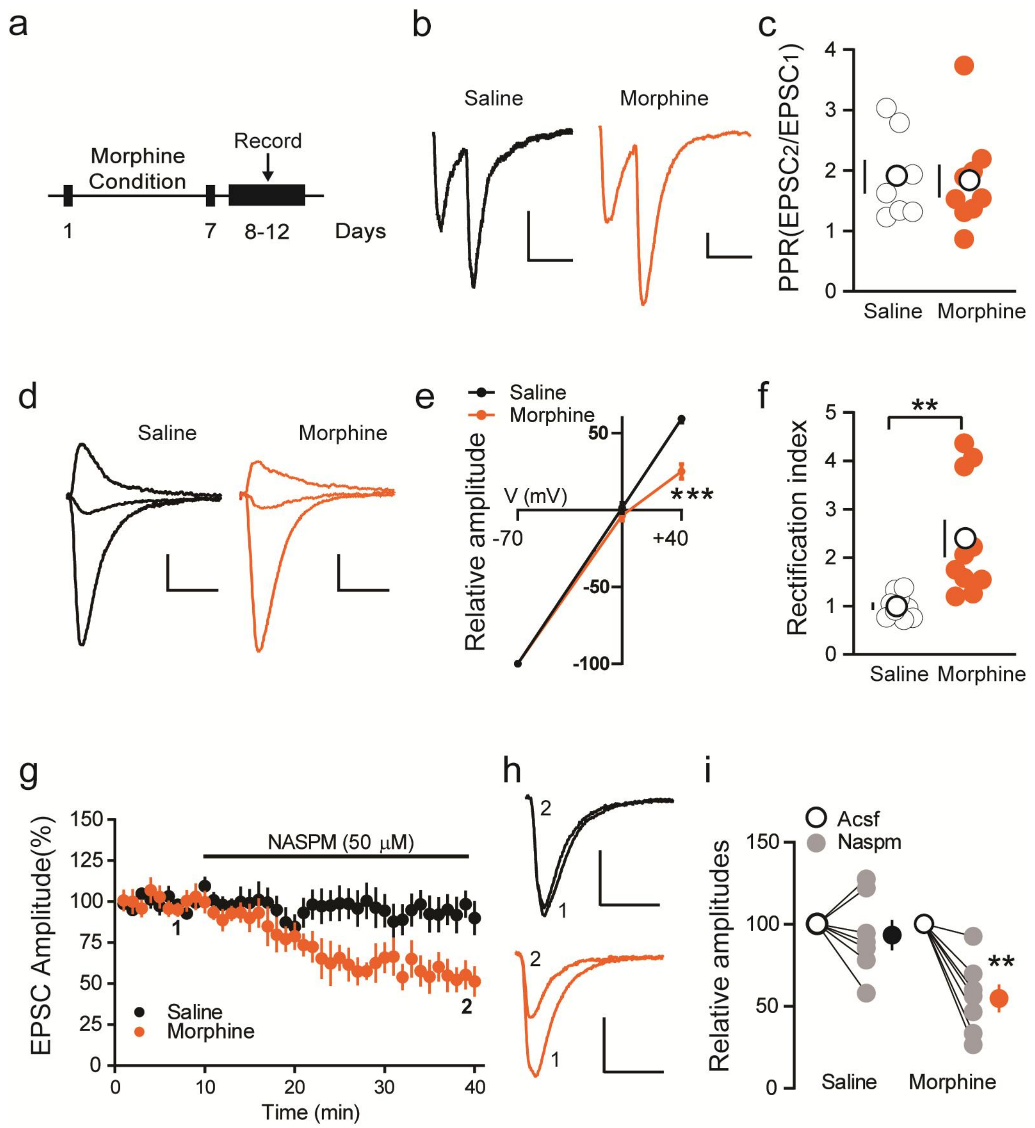

Figure S2. CP-AMPARs contributed to morphine-induced synaptic potentiation in PL-mPFC layer V pyramidal neurons. a, Experimental timeline of electrophysiological recording. b,c, Example traces (b) and quantification (c) of paired-pulse ratio show that morphine conditioning did not affect transmitter release probability in PL-mPFC (saline/morphine, n=6/9). unpaired t test. $p > 0.05$. Scale bar: 50 pA, 25 ms. d-f, Example traces (d), I/V curve (e) and quantification (f) of evoked AMPAR EPSCs at −70 mV, 0 mV and +40 mV show that the rectification in morphine group (saline/morphine, n=10/10). Two-way ANOVA ($F (1, 30) = 3.697$, $p < 0.001$) followed by Post-hoc Sidak's test. ***$p < 0.001$. Unpaired t test. *$p < 0.05$. Scale bar: 50 pA, 50 ms. g-i, Time course (g), example traces (h) and quantification (i) of EPSCs (−70 mV) before (black outlined)

and during (gray filled) perfusion of Naspm (50 µM) show a significantly increased presence of synaptic CP-AMPAR (saline/morphine, n=7/7). Unpaired t test. $**p < 0.01$. Scale bar: 50 pA, 25 ms. Mean ± s.e.m.

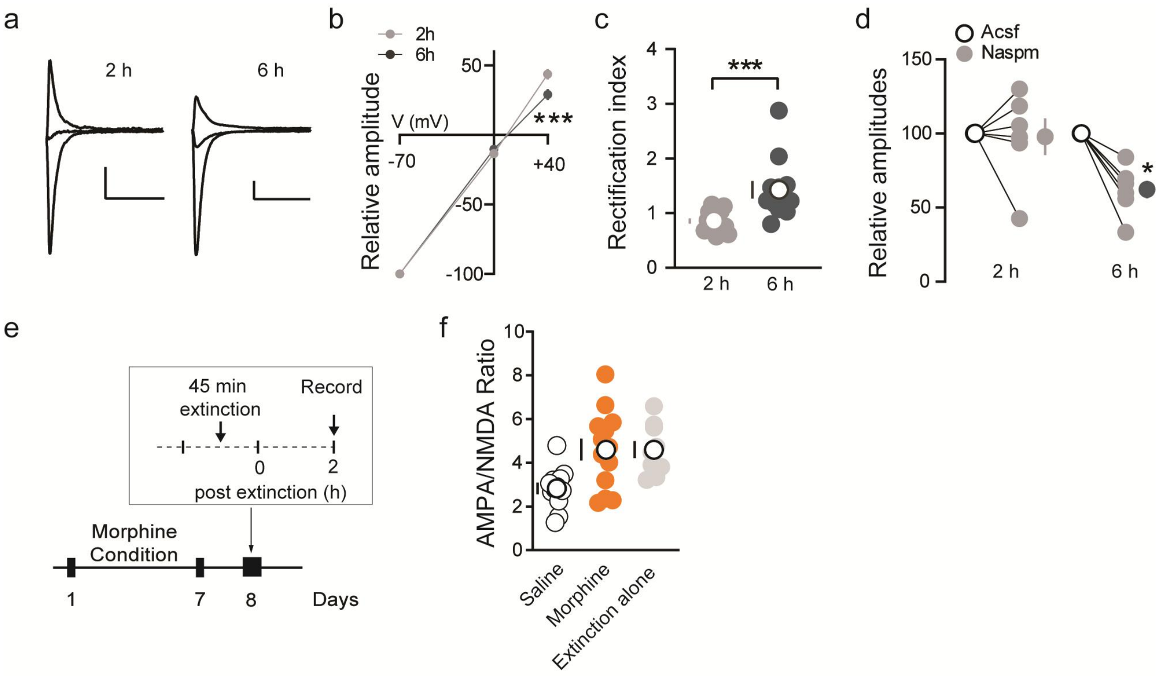

Figure S3. Calcium-permeable AMPA Receptor dynamics during retrieval. a-c, Example traces (a), I/V curve (b) and quantification (c) of evoked AMPAR EPSCs at −70 mV, 0 mV and +40 mV show that the rectification was abolished 2 h post retrieval while recover ~6 h post retrieval (2 h/6 h, n=15/12). Two-way ANOVA ($F (1, 75) = 2.772$, $p < 0.001$) followed by Post-hoc Sidak's test. $***p < 0.001$. Unpaired t test. $***p < 0.001$. Scale bar: 50 pA, 50 ms. g, Quantification of EPSCs before (black outlined) and during (gray filled) perfusion of Naspm (50 µM) show a significantly decreased presence of synaptic CP-AMPAR 2 h post retrieval while increased 6 h post (2 h/6 h, n=6/7). Unpaired t test. $*p < 0.05$. Mean ± s.e.m. e. Experimental timeline. f, Quantification of evoked EPSCs at -70 and +40 mV recording from extinction alone mice show extinction alone did not induce a decrease of synaptic strength 2 h post extinction (saline/morphine/extinction alone, n=12/13/9). One-way ANOVA ($F (2, 31) = 3.018$, saline vs morphine, $p < 0.01$; saline vs extinction alone, $p < 0.05$) followed by Dunnett's test. $*p < 0.05$, $**p < 0.01$.data of saline and morphine group from figure 2b.

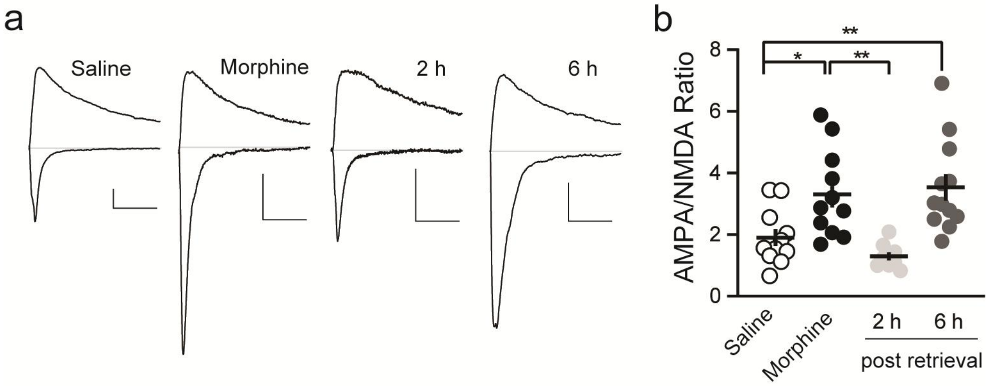

Figure S4. Retrieval induced synaptic remodeling in PL-mPFC of female mice. a, b, Example traces (a) and quantification (b) of evoked EPSCs at -70 and +40 mV recording from saline, morphine (without retrieval), 2 h, 6 h post retrieval group mice show an increase of AMPA/NMDA ratio after morphine condition and a quick decrease and recover ~6 h post retrieval in mPFC of female mice (saline/morphine/2 h/ 6 h, n=11/11/9/12). One-way ANOVA ($F_{(3, 39)} = 8.374$, saline vs morphine, $p < 0.05$; saline vs 6 h, $p < 0.01$; morphine vs 2 h, $p < 0.01$) followed by Dunnett's test. *$p < 0.05$, **$p < 0.01$. Scale bar: 50 pA, 50 ms.

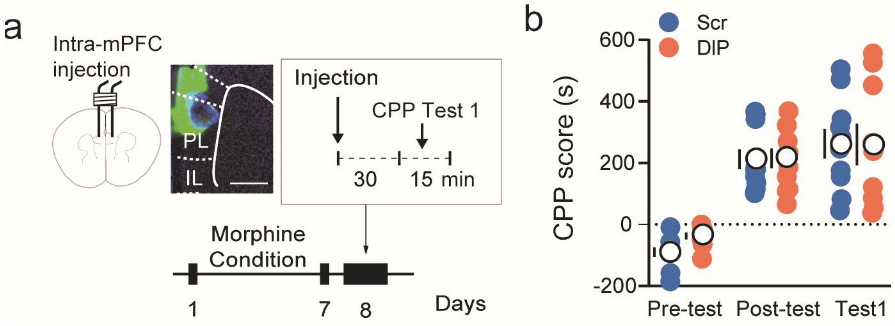

Figure S5. Preventing CP-AMPAR dynamics during synapse remodeling did not affect CPP expression. a, Experimental timeline of behavior test. b, SCH23390 treatment did not affect CPP expression (saline/SCH, n=10/9). Two-way ANOVA (F (1,51) = 0.3036, $p > 0.05$) followed by Post-hoc Sidak's test. Mean ± s.e.m.

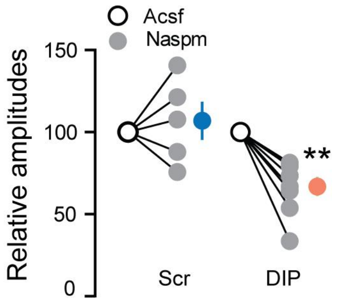

Figure S6. Preventing retrieval-induced synapse remodeling blocked the intervention efficacy of retrieval-extinction procedure on CP-AMPARs.
Quantification of EPSCs before (black outlined) and during (gray filled) perfusion of Naspm (50 μM) show a significantly increased synaptic CP-AMPAR components in DIP group (Scr/DIP, n=5/8). Unpaired t test. ** $p < 0.01$. Scr, scramble peptide; DIP, dynamin inhibitory peptide. Mean ± s.e.m.

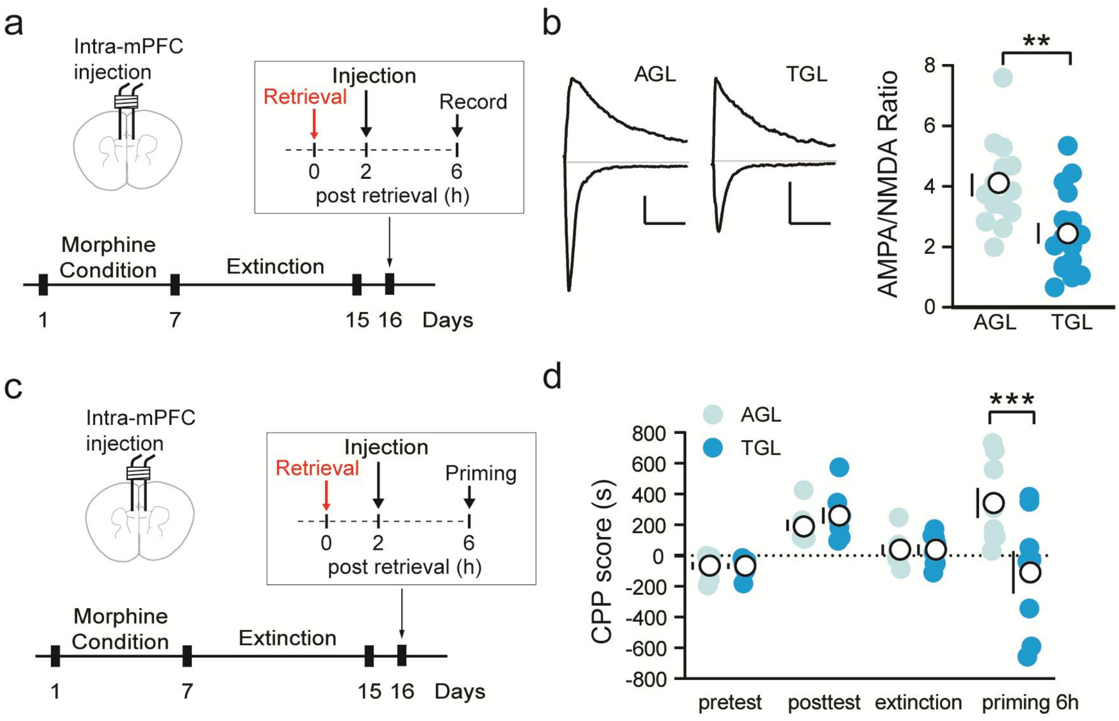

Figure S7. Reducing PL synaptic strength impaired reinstatement. a. Experimental timeline of behavior and recording. b, Example traces (left) and quantification (right) of evoked EPSCs at -70 and +40 mV show intra-mPFC TGL injection prevented re-strengthening of remodeling synapse (AGL/TGL, n=14/16). Unpaired t test. ***p* < 0.01. Scale bar: 50 pA, 50 ms. c, Experimental timeline of behavior and recording of d. d, Effect of the experimental manipulations on the CPP score show drug-priming-induced reinstatement was significantly decreased in TGL group (AGL/TGL, n=8/8). Two-way ANOVA (F (1, 56) = 4.147, $p < 0.001$) followed by Post-hoc Sidak's test. *** $p < 0.001$. Mean ± s.e.m

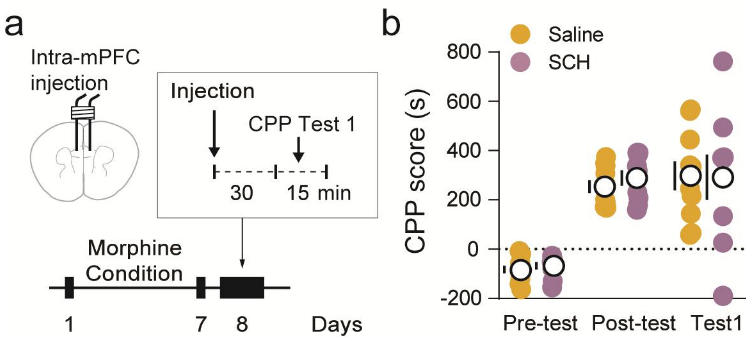

Figure S8. Inhibition of dopamine D1 receptors during synapse remodeling did not affect CPP expression. a, Experimental timeline of b. b, SCH23390 treatment did not affect CPP expression (saline/SCH, n=10/9). Two-way ANOVA ($F(1, 51) = 0.1443$, $p > 0.05$) followed by Post-hoc Sidak's test. SCH, SCH23390; Mean ± s.e.m.

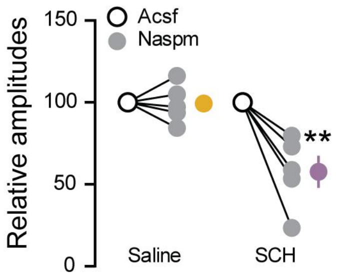

Figure S9. Suppression D1R signaling blocked the intervention efficacy of retrieval-extinction procedure procedure on CP-AMPARs. Quantification of EPSCs before (black outlined) and during (gray filled) perfusion of Naspm (50 μM) show a significantly increased synaptic CP-AMPAR components in SCH group (Saline/SCH, n=5/5). Unpaired t test. **p < 0.01. SCH, SCH23390. Mean ± s.e.m.
